# Effect of Genetic Variation in *STXBP5* and *STX2* on von Willebrand Factor and Bleeding Phenotype in Type 1 von Willebrand Disease Patients

**DOI:** 10.1371/journal.pone.0040624

**Published:** 2012-07-06

**Authors:** Janine E. van Loon, Yvonne V. Sanders, Eva M. de Wee, Marieke J. H. A. Kruip, Moniek P. M. de Maat, Frank W. G. Leebeek

**Affiliations:** Department of Haematology, Erasmus Medical Center, Rotterdam, The Netherlands; Institut National de la Santé et de la Recherche Médicale, France

## Abstract

**Background:**

In type 1 von Willebrand Disease (VWD) patients, von Willebrand Factor (VWF) levels and bleeding symptoms are highly variable. Recently, the association between genetic variations in *STXBP5* and *STX2* with VWF levels has been discovered in the general population. We assessed the relationship between genetic variations in *STXBP5* and *STX2*, VWF levels, and bleeding phenotype in type 1 VWD patients.

**Methods:**

In 158 patients diagnosed with type 1 VWD according to the current ISTH guidelines, we genotyped three tagging-SNPs in *STXBP5* and *STX2* and analyzed their relationship with VWF:Ag levels and the severity of the bleeding phenotype, as assessed by the Tosetto bleeding score.

**Results:**

In *STX2*, *rs*7978987 was significantly associated with VWF:Ag levels (bèta-coefficient (β) = −0.04 IU/mL per allele, [95%CI −0.07;−0.001], p = 0.04) and VWF:CB activity (β = −0.12 IU/mL per allele, [95%CI −0.17;−0.06], p<0.0001). For *rs*1039084 in *STXBP5* a similar trend with VWF:Ag levels was observed: (β = −0.03 IU/mL per allele [95% CI −0.06;0.003], p = 0.07). In women, homozygous carriers of the minor alleles of both SNPs in *STXBP5* had a significantly higher bleeding score than homozygous carriers of the major alleles. (Rs1039084 p = 0.01 and rs9399599 p = 0.02).

**Conclusions:**

Genetic variation in *STX2* is associated with VWF:Ag levels in patients diagnosed with type 1 VWD. In addition, genetic variation in *STXBP5* is associated with bleeding phenotype in female VWD patients. Our findings may partly explain the variable VWF levels and bleeding phenotype in type 1 VWD patients.

## Introduction

Von Willebrand factor is a multifunctional glycoprotein, which is involved in platelet adhesion and subsequent platelet aggregation during primary haemostasis [1], [2]. Low VWF levels are a diagnostic criterion for Von Willebrand Disease (VWD), the most common inherited bleeding disorder. VWD is classified as either a quantitative deficiency of von Willebrand Factor (VWF) (type 1 and 3 VWD) or as a qualitative defect of VWF molecules (type 2 VWD). Furthermore, according to the current ISTH guidelines diagnosis of VWD is based on clinical criteria, including a mucocutaneous bleeding history and a family history of excessive bleeding [3].

In type 1 VWD patients, who have reduced but functionally normal VWF molecules, VWF levels are highly variable. Also, the VWD phenotype penetrates incompletely leading to a variable clinical presentation. To date, we can only explain part of the variation in VWF levels and bleeding symptoms in these patients.

In recent years, genome-wide association studies have been performed and have enabled us to investigate the genetic component of common diseases and quantitative traits without a prior biological hypothesis. In this way, novel genetic determinants of VWF:Ag levels have been discovered: *STXBP5*, *SCARA5*, *STAB2*, *STX2*, *TC2N*, and *CLEC4M* [4]. Our interest was especially focussed on STXBP5 and STX2, because their encoding proteins are suggested to be involved in WPB exocytosis [5].

Syntaxin 2 (*STX2*) is a binding substrate for the Syntaxin Binding Protein 5 (*STXBP5*) and is a member of the Soluble N-ethylmaleimide-sensitive factor (NSF) Attachment protein Receptor (SNARE) protein family. These proteins drive vesicle exocytosis by fusion of granules and target membranes, a process involved in the regulation of numerous secretory events [6]. STXBP5 and STX2 interact specifically with SNARE complex proteins, such as SNAP23 and syntaxin-4. These proteins have been shown to be involved in Weibel Palade Body (WPB) exocytosis, the well known mechanism for the secretion of VWF molecules by endothelial cells [5].

Dysfunction of the WPB machinery is a likely contributor to the variation in VWF:Ag levels in type 1 VWD patients. Also, since we have recently confirmed the association between genetic variation in *STXBP5* and *STX2* and VWF:Ag levels in young patients with a first event of arterial thrombosis [7], we hypothesized that genetic variation in *STXBP5* and *STX2* may also affect VWF:Ag levels in patients diagnosed with type 1 VWD according to the current ISTH guidelines. In addition, genetic variation in *STXBP5* and *STX2* may, by regulating VWF:Ag levels, influence the incomplete penetrance and the variable clinical presentation of type 1 VWD. Therefore, we aimed to assess the relationship between genetic variation in *STXBP5* and *STX2*, VWF:Ag levels, and the bleeding phenotype in patients previously diagnosed with type 1 VWD.

## Methods

### Study Population

In this study we included 158 patients, who were previously diagnosed with type 1 VWD in the Erasmus University Medical Center Rotterdam. The diagnosis of type 1 VWD was based on clinical bleeding symptoms, a family history of bleeding, and VWF:Ag levels or VWF:Rco activity ≤0.30 IU/mL, according to the current ISTH guidelines [3].

### Ethics Statement

The study was approved by the medical research board at Erasmus University Medical Center and written informed consent was obtained from all participants at inclusion.

### Laboratory measurements

Blood was drawn by venipuncture in the antecubital vein using Vacutainer system (Becton-Dickinson, Plymouth, UK). Blood for coagulation measurements was collected in 3.2% trisodium citrate (9∶1 vol/vol). Citrated blood was centrifuged within 1 hour at 2000×g for 10 min at 4°C. Plasma was additionally centrifuged at 14,000×g for 10 minutes at 4°C and stored in aliquots at −80°C. For DNA isolation blood was collected in tubes containing ethylene diaminetetraacetic acid (EDTA; Beckon Dickinson) and genomic DNA was isolated according to standard salting-out procedures and stored at 4°C for genetic analysis.

VWF:Ag was determined with an in-house ELISA with polyclonal rabbit anti-human VWF antibodies and horseradish peroxidase conjugated anti-human VWF antibodies (DakoCytomation, Glostrup, Denmark) for catching and tagging, respectively. For our analyses we used the lowest VWF:Ag level that was ever measured in a patient (historical VWF:Ag measurement).

VWF collagen binding (VWF:CB) was measured with an in-house ELISA using type I collagen (Sigma, St. Louis, USA) for catching and horseradish peroxidase conjugated anti-human VWF antibodies for tagging.

VWF:RCo was assayed with formalin-fixed platelets using a PAP4 Model Aggregometer (Bio-Data Corp. Hatboro, Pennsylvania) according to Macfarlane et al. [8].

### Bleeding score

The Bleeding Score was used as previously described for bleeding severity in type 1 VWD by Tosetto et al [9]. It systematically evaluates bleeding symptoms, and accounts for both the number and severity of the bleeding symptoms. Each patient completed a questionnaire, which included the Bleeding Score assessment tool. The severity and frequency of twelve items are scored on a scale ranging from minus one to four points. Higher scores reflect a more severe bleeding phenotype characterized by more severe or more frequent bleeding. The total for all twelve items result in a Bleeding Score (range −3 to 45).

### Genotyping analysis

The *STXBP5* gene spans 182 kbps and is located in the q24 region of chromosome 6. The *STX2* gene spans 50 kbp and is located in the q24.3 region of chromosome 12. Initially, we obtained data from the International HapMap project (phase II November 2008 http://www.hapmap.org) on the linkage disequilibrium (LD) pattern and selected haplotype-tagging single-nucleotide polymorphisms (ht-SNPs) using Haploview software (version 3.11; www.broad.mit.edu/mpg/haploview/index/php). For both genes blocks of haplotypes with a frequency of ≥3% were defined in order to select ht-SNPs. We took potential functionality into consideration by preferentially selecting non-synonymous ht-SNPs or SNPs that are located in known regulatory elements. We considered only SNPs that were present in a Caucasian population. Of these ht-SNPs, three were significantly associated with VWF:Ag levels in our previous study among young patients with arterial thrombosis and healthy controls [7]. Therefore, we selected and genotyped only these three SNPs in *STXBP5* (*rs*1039084 and *rs*9399599) and in *STX2* (*rs*7978987) for our current study using Custom TaqMan Genotyping Assays (Applied Biosystems, Foster City, CA, USA). The polymorphisms in *STXBP5*, rs1039084 and rs9399599, are in high linkage disequilibrium with *rs*9390459, which had the highest genome wide significance level for VWF plasma levels in the meta-analysis of the CHARGE consortium (D′ = 1.00, R^2^ = 0.87 for *rs*9399599 and D′ = 0.96, R^2^ = 0.86 for *rs*1039084) (phase II November 2008 http://www.hapmap.org). Also, rs7978987 in *STX2* had a highly significant *P* value of 3.82×10^−11^ in this meta-analysis. Endpoint fluorescence was measured on the ABI 7900HT instrument (Applied Biosystems, Foster City, CA, USA) and clustered according to genotype using SDS 2.1 software (Applied Biosystems, Foster City, CA, USA). Genotyping was successful for each SNP in on average 96% of all subjects.

### Statistical analysis

Allele frequencies were calculated by genotype counting. For each SNP the deviation from the Hardy-Weinberg equilibrium was tested by means of a Chi-squared test with one degree of freedom. VWF:Ag, VWF:RCo, and VWF:CB levels per genotype of each SNP were assessed by analysis of covariance (ANCOVA) adjusted for age and sex. Homozygous carriers of the VWF:Ag increasing allele were used as reference category. In addition, to analyze the trend across genotypes we performed linear regression analysis on VWF measures using the genotypes of each SNP as a continuous variable under the assumption of an additive genetic model (i.e. alleles have no dominant or recessive effect). To investigate the effect of ABO blood group on this association, the latter analysis was additionally adjusted for blood group (O and non-O).

The effect of genetic variation on the bleeding score has been assessed by ANCOVA adjusted for the age at the time of completing the questionnaire. Since the bleeding score differs significantly between men and women, we stratified this analysis by sex additionally.

Statistical analyses were performed with SPSS for Windows, version 17.0 (SPSS Inc, Chicago, USA). A two-sided value of p<0.05 was considered statistically significant.

## Results

### Baseline characteristics

Baseline characteristics of the study population are displayed in table 1. We included 158 subjects diagnosed with type 1 VWD, who had a mean historical VWF:Ag level of 0.35±0.13 IU/mL (mean ± standard deviation), VWF:CB of 0.29±0.13 IU/mL, and VWF:Rco of 0.27±0.14 IU/mL. The mean age at inclusion was 46.0±16.8 years and 100 patients (63.3%) were female. Blood group O was the most frequent among these patients (78.5%). The allele frequency distributions of the genetic polymorphisms did not deviate from the Hardy–Weinberg equilibrium.

**Table 1:** Baseline characteristics of the study population.

	Type 1 VWD patients	Males	Females	P value
	(N = 158)	(N = 58)	(N = 100)	
Age (years)	46.0±16.8	42.9±19.4	47.8±14.9	0.08
Female (%)	100 (63.3%)	-	-	
Blood group O (%)	124 (78.5%)	46 (79.3%)	78 (78.0%)	0.85
Bleeding score (points)	11±7	8±6	12±7	0.001
VWF:Ag (IU/mL)	0.35±0.13	0.34±0.11	0.36±0.14	0.38
VWF:CB (IU/mL)	0.29±0.21	0.28±0.16	0.30±0.24	0.64
VWF:RCo (IU/mL)	0.27±0.14	0.26±0.11	0.28±0.16	0.48

Summary statistics for continuous variables are presented as mean ± standard deviation. Categorical data are summarized as percentages. Abbreviations used in this table are VWF for Von Willebrand Factor and VWD for Von Willebrand Disease. The P value represents the difference between sexes for each variable.

### Association between polymorphisms in STXBP5 and STX2 and VWF:Ag levels

In *STX2*, *rs*7978987 (intron 9) was significantly associated with VWF:Ag levels (beta-coefficient (β) = −0.04 IU/mL per allele, [95%CI −0.07;−0.001], p = 0.04) and VWF:CB activity (β = −0.12 IU/mL per allele, [95%CI −0.17;−0.06], p<0.0001), also after adjustment for blood group (p = 0.04 for VWF:Ag and p<0.0001 for VWF:CB) (table 2). Interestingly, the frequency of the minor allele (MAF) of rs7978987, which corresponded with higher VWF:Ag levels, was lower among our patients with type 1 VWD (MAF = 0.29) than reported by dbSNP (http://www.ncbi.nlm.nih.gov/projects/SNP/) (MAF = 0.38) and in the meta-analysis of the CHARGE consortium (MAF = 0.35) [4].

**Table 2:** VWF:Ag, VWF:RCo, and VWF:CB per genotype.

SNP#	Gene	N	VWF:Ag	VWF:RCo	VWF:CB
			(IU/mL)	(IU/mL)	(IU/mL)
rs1039084	*STXBP5*				
GG		37	0.38±0.02	0.30±0.02	0.33±0.04
AG		79	0.35±0.02	0.27±0.02	0.31±0.03
AA		34	0.33±0.02	0.25±0.03	0.25±0.04
*p for trend*			0.07	0.09	0.12
rs9399599	*STXBP5*				
AA		36	0.36±0.02	0.28±0.02	0.33±0.04
AT		76	0.35±0.02	0.27±0.02	0.28±0.03
TT		39	0.34±0.02	0.25±0.02	0.30±0.04
*p for trend*			0.40	0.31	0.62
rs7978987	*STX2*				
AA		10	0.40±0.04	0.28±0.05	0.53±0.07
AG		67	0.37±0.02	0.28±0.02	0.32±0.03
GG		73	0.33±0.02	0.26±0.02	0.24±0.03
*p for trend*			0.04	0.43	<0.0001

VWF:Ag, VWF:Rco, and VWF:CB levels (mean ± SE) per genotype of each SNP (ANCOVA adjusted for age and sex). P for trend was calculated using linear regression on VWF:Ag measures with additive genetic models. Abbreviations used in this table are SNP for single nucleotide polymorphism, MAF for minor allele frequency, and VWF for Von Willebrand Factor.

**Figure 1:**
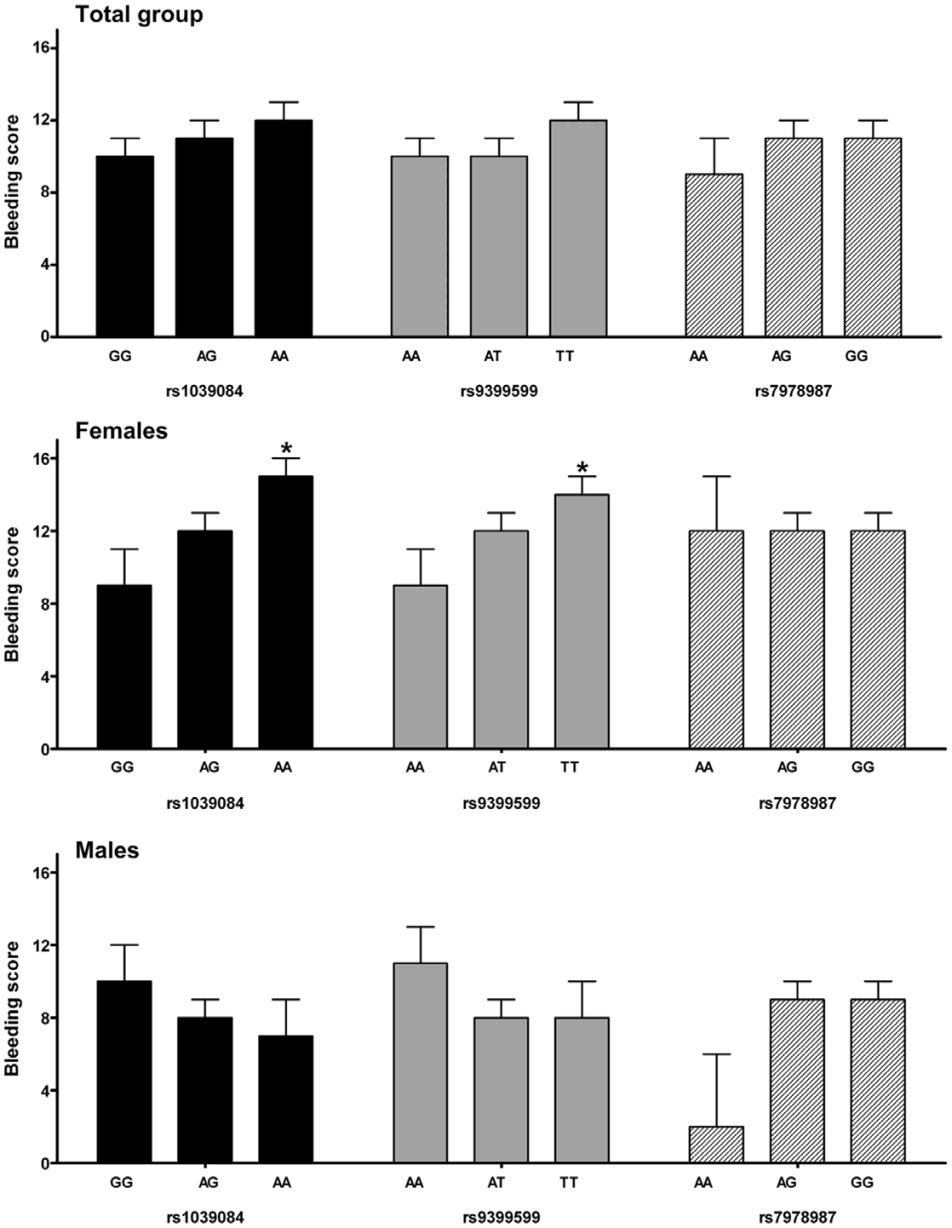
Haplotype analysis for rs1039084 and rs9399599. Graph presents the coefficients with standard error per haplotype. Haplotype 1 was used as reference haplotype. NS = not significant.

For *rs*1039084 in *STXBP5*, which is a missense mutation that encodes an amino acid substitution of asparagine into serine at position 436 a similar trend with VWF:Ag levels was observed: (β = −0.03 IU/mL per allele [95% CI −0.06;0.003], p = 0.07) (table 2). *Rs*9399599 in STXBP5, which is located in intron 25, was not associated with VWF:Ag levels nor with VWF:CB, though carriers of the minor allele had lower VWF:Ag levels and VWF:CB activity as was also observed for rs1039084. Rs1039084 and rs9399599 have a strong linkage disequilibrium of D′ = 0.88 and R^2^ = 0.71. Haplotype analysis showed that both SNPs contribute to the variation in VWF:Ag levels (figure 1).

The VWF:RCo activity and VWF:CB activity showed similar associations for both SNPs in STXBP5, although not statistically significant (table 2).

### Association between polymorphisms in STXBP5 and STX2 and the bleeding phenotype

**Figure 2:**
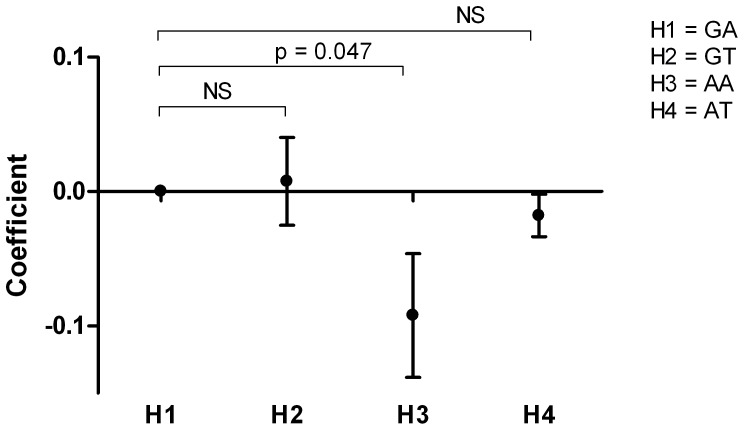
Bleeding score. Bleeding score per genotype for each SNP for the total group and stratified by sex. * P<0.05.

The mean bleeding score was 12±8 in women (N = 100) and 8±6 in men (N = 58) (P = 0.001). In women, homozygous carriers of the minor alleles of both SNPs in *STXBP5* had a significantly higher bleeding score than homozygous carriers of the major allele (rs1039084: GG 9±2 versus AA 15±2, p = 0.01 and rs9399599: AA 9±2 versus TT 14±1, p = 0.02) (figure 2). Rs7978987 in *STX2* was not associated with the bleeding score.

## Discussion

In the current study we have shown that genetic variation in STX2 and to a lesser extent in *STXBP5* contributes to VWF:Ag levels in patients diagnosed with type 1 VWD. In addition, we demonstrate that in women with type 1 VWD genetic variation in *STXBP5* is associated with the bleeding phenotype.

We are the first to describe the association between genetic variation in SNARE protein genes and VWF:Ag levels and VWF:CB in patients previously diagnosed with type 1 VWD. In our analysis we used historical levels, since we expect that the lowest levels ever measured are the least influenced by external factors, such as inflammation, hormones, and stress, and therefore reflect a steady state situation.

SNPs in *STXBP5* and *STX2* have been previously identified in the meta-analysis of the CHARGE consortium as determinants of VWF levels in the general population. The effect sizes we obtained were comparable with previous studies [4], [7]. In our study, the selected SNPs are the same or are in high linkage disequilibrium with those identified in the CHARGE meta-analysis. Though the selected SNPs in *STXBP5* are in high LD (D′ = 0.88 and R^2^ = 0.71) the effect estimate of rs1039084 was slightly higher than for rs9399599. Haplotype analysis showed that both SNPs have an effect on VWF:Ag levels. It can be anticipated that since rs1039084 is a non-synonymous SNP and rs9399599 is intronic, rs1039084 may be the actual causal variant with a greater effect.

Interestingly, the frequency of the minor allele of rs7978987 that was significantly associated with higher VWF:Ag levels, was much lower in our type 1 VWD patients (MAF = 0.29) than reported by dbSNP (MAF = 0.38) and the CHARGE consortium (MAF = 0.35). Since the patients were selected on their VWF:Ag levels, it was expected that the frequency of genetic variants associated with higher VWF:Ag levels was much lower in our population.

Alongside the association between rs7978987 and VWF:Ag levels, this SNP was also significantly and more strongly associated with VWF:CB. Since VWF:CB is related to the multimer size of VWF and WPBs contain ultra-large VWF molecules only, this finding may point to actual involvement of *STX2* in WPB exocytosis. However, this hypothesis can not be further substantiated and should await future research.

One striking finding is the association between genetic variation in *STXBP5* and the bleeding phenotype, as measured by the bleeding score, in women with type 1 VWD. In the total population and in men we did not observe this association. This can be explained by the fact that women experience generally more challenges to the haemostatic system during life, whereby the bleeding score in women may be a better reflector of clinically relevant bleeding tendency, than in men. Indeed, we observed that the menorraghia item of the bleeding score mainly drives the association between genetic variation in STXBP5 and the bleeding score. Nevertheless, the association with bleeding score, which was assessed by a self-administrated questionnaire, should be interpreted with caution and replicated in larger cohorts, since the number of women per genotype was low.

The association between genetic variation in *STX2* and VWF:Ag levels was independent of blood group. Blood group is the most important determinant of VWF:Ag levels. The presence of blood group A and B antigens on VWF molecules leads to a decreased clearance of VWF molecules. Consequently, individuals with blood group O have approximately 25% lower VWF plasma concentrations than individuals with blood group non-O [10]. Furthermore, as blood group O corresponds with lower VWF:Ag levels, patients diagnosed with type 1 VWD more frequently have blood group O than non-O. We adjusted our statistical analysis for blood group, but this did not influence the effect size of the association. This finding meets our expectations, since we included only moderate and severe type 1 VWD. Also, genetic variation in *STXBP5* and *STX2* may affect the release of VWF molecules, which is expected to be similar in subjects with blood group O and in subjects with blood group non-O, rather than the clearance.

In today's clinical practice diagnosis of type 1 VWD is difficult, because of the high variability in VWF plasma levels and the incomplete penetrance of the phenotype. In addition, there is only a weak association between low VWF:Ag levels and bleeding symptoms, which are both highly frequent in the general population. For these reasons it is sometimes hard to distinguish between physiologically low VWF levels and low VWF levels because of type 1 VWD [11]. This problem is further underlined by the fact that a substantial number of individuals with low VWF levels have no clear family history of bleeding symptoms and no detectable mutations in the VWF gene (*VWF*), although is has been anticipated for a long time that type 1 VWD is caused by *VWF* mutations. Three large studies in Europe, the United Kingdom and Canada showed that only 65% of the type 1 VWD patients have candidate mutations, meaning that 35% have no apparent *VWF* mutations [12], [13], [14]. Therefore it is likely that genetic variations in genes other than *VWF* may contribute to the variation in VWF levels and bleeding symptoms, as we present in the current study.

Our study had a few limitations. First, we have not investigated the combined effect of both SNARE protein gene variations and *VWF* mutations on VWF:Ag levels, since we have not performed VWF gene mutation analysis in our cohort. However, we expect that mutations in the VWF gene do not interact with polymorphisms in *STXBP5* and *STX2*, since *STXBP5* is located on a different chromosome than *VWF* and *STX2* is located more than 120.000 kb from the VWF gene. Therefore, *VWF* mutations will be equally distributed across the genotypes. Finally, we are aware that our sample size is small and that our findings require replication in larger cohorts of patients with type 1 VWD. However, our findings are innovative and form the basis for further research on the role of SNARE protein genes in the clinical presentation of type 1 VWD.

In conclusion, we have shown that genetic variation in *STX2* is associated with VWF:Ag levels in patients previously diagnosed with type 1 VWD. In addition, genetic variation in *STXBP5* is associated with the bleeding phenotype in female type 1 VWD patients. Our findings may explain part of the variation in VWF levels and bleedings symptoms in patients with type 1 VWD. Also, alongside known VWF mutations, genetic variations in SNARE protein genes may help to diagnose individuals with low VWF levels in the future.
